# Predicting Longitudinal Progression in Functional Mobility After Stroke: A Prospective Cohort Study

**DOI:** 10.1161/STROKEAHA.120.029913

**Published:** 2020-06-17

**Authors:** Dongni Buvarp, Lena Rafsten, Katharina S. Sunnerhagen

**Affiliations:** 1 Rehabilitation Research Group, Department of Clinical Neuroscience, Institute of Neuroscience and Physiology (D.B., L.R., K.S.S.), University of Gothenburg, Sweden.; 2 Sahlgrenska University Hospital, Gothenburg, Sweden (L.R., K.S.S.).

**Keywords:** cluster analysis, follow-up, gait, stroke, time factors

## Abstract

A majority of people with stroke remain impaired in their functional mobility. The aim of the study was to determine longitudinal changes in functional mobility after stroke.

**Methods:**

The study was of a longitudinal and prospective design. The functional mobility was assessed using the Timed Up-and-Go test on 5 different occasions: 5 days after onset, within 24 hours after discharge, 1 month after discharge, 3 months, and 1 year poststroke. Stroke severity was stratified based on impairments and activity limitations using a baseline cluster analysis. A multilevel model was developed to predict longitudinal progression in functional mobility based on stroke severity.

**Results:**

One-hundred-thirty-five patients were included at baseline. Two distinct subgroups (moderate stroke [52%] and mild stroke [48%]) were identified based on impairments and activity limitations using clustering. Ninety-one patients were included in the longitudinal analysis. After controlling for age and cognition, significant improvements in functional mobility were found in the moderate stroke group between baseline and 1 year poststroke (mean difference in Timed Up-and-Go test time, –6.4 s, adjusted *P*<0.001). Patients with moderate stroke had a maximum rate of improvement in functional mobility during the first 3 months poststroke and then declined significantly at 1 year (12% increase in Timed Up-and-Go test time, adjusted *P*=0.025). Younger patients had better functional mobility. Although there was also a slight improvement in the mild stroke group, it was not statistically significant.

**Conclusions:**

The recovery of functional mobility differs between mild and moderate stroke. Patients with moderate stroke improved their functional mobility during the first 3 months, after which it decreased significantly. These findings suggest that long-term rehabilitation is desirable to maintain and perhaps increase the gained functional mobility. Older patients and those with moderate impairments and activity limitations have particular needs.

**Registration:**

URL: https://www.clinicaltrials.gov. Unique identifier: NCT01622205.

## Abstract 2

Supplemental Digital Content is available in the text.

Stroke is a leading cause of long-term disability worldwide.^1^ A decline in functional mobility is a widely recognized residual impairment after stroke and refers to the ability to transfer (eg, getting in and out of a bed or a chair), the ability to walk a certain distance and turn, and is associated with maintaining independence. Inactivity and social isolation caused by decreased functional mobility have a significant decline in the quality of life in patients with stroke. This may lead to a higher morbidity and mortality rate.

Regaining the ability to walk independently and recovery of mobility have been identified as highly significant by patients after stroke.^2^ Older age,^3,4^ inactivity,^5^ and cognitive impairment^5,6^ were shown to be predictors of functional mobility decline. However, it remains unclear how the recovery of functional mobility varies among patients with different severities of impairments and activity limitations—for example, whether the individuals with mild paresis recover their functional mobility faster.

Longitudinal studies are warranted as impaired functional mobility is considered to be a major problem resulting in falls and dependency after stroke. The recovery of functional mobility is of high clinical interest in relation to implementing and planning rehabilitation. Modeling individual changes and time effect, especially nonlinear time effect, on stroke recovery has been stressed as valuable in recovery prediction.^7^ Better knowledge of the longitudinal progression in functional mobility can be used to guide clinical management after stroke to provide the right rehabilitation to the right person at the right time. Knowledge of longitudinal changes would also allow more insight into underlying mechanisms of recovery. The aim of the study was to investigate longitudinal progression in functional mobility during the first year of stroke and examine whether the rate of change in functional mobility differs between different levels of stroke severity.

## Methods

### Data and Material Availability

According to the Swedish regulations shown in https://etikprovning.se/for-forskare/ansvar/, the complete dataset cannot be made publicly available for ethical and legal reasons. Researchers can request access to the data by emailing the principal investigator at ks.sunnerhagen@neuro.gu.se.

### Study Population

The study population was enrolled in the Gothenburg Very Early Supported Discharge study trail at Sahlgrenska University Hospital, Sweden from September 2011 to April 2016. Eligible participants were age >18 and had a diagnosis of stroke according to World Health Organization criteria.^8^ Exclusion criteria were as follows: if the participants were living >30 minutes traveling time to the hospital; National Institute of Health Stroke Scale score >16; Barthel Index (BI) <50 (severe functional impairment) and life expectancy less than 1 year (eg, malignant disease). Full inclusion and exclusion criteria were reported earlier in detail.^9^ The study protocol was approved by the Regional Ethical Review Board in Gothenburg, Sweden. Written informed consent was obtained from all the participants, in agreement with the declaration of Helsinki.

### Study Design

The study was of longitudinal and prospective design. Functional mobility was assessed using the Timed up-and-Go test (TUG) was conducted across the following occasions: 5 days after onset, within 24 hours after discharge, 1 month after discharge, and 3 months and 1-year poststroke. TUG is a well-validated objective instrumental test for assessing functional mobility.^10^ It measures the time it takes for participants to rise from an armchair, walk 3 meters at their own paces, turn around, walk back to the chair, and sit down.^10^ TUG was performed twice on each occasion and the second test value was used. A shorter TUG time indicates better functional mobility. The number of steps taken during the assessment was also recorded. A TUG time of ≤10 s is interpreted as normal functional mobility in clinical practice,^11^ while a time of 20 s suggests a need for assistance in daily living, and a time of ≥30 s indicates immobility and that a walking aid is required.^11^

Clinical assessments including impairments and activity limitations were conducted by an experienced physiotherapist or occupational therapist. Neurological deficit severity was assessed using the National Institute of Health Stroke Scale at 2 days after stroke.^12^ The Fugl-Meyer Assessment was used to assess motor function, sensation, passive range of joint motion, and joint pain of the upper and lower extremities.^13^ A higher Fugl-Meyer Assessment score indicates better sensory-motor function. Cognitive function was assessed using the Montreal Cognitive Assessment, scored from 0 to 30 (higher indicates better cognition).^14^ Overall disability was assessed using the modified Rankin Scale, which consists of an ordinal scale ranging from 0 to 6 (0 corresponds to no symptoms at all, 5 to severe disability and 6 to death).^15^ Dependency in daily activities of living was assessed using the 10-item ordinal BI, with a range of scores from 0 to 100 (higher indicates greater independence).^16^ All these measurements can be used to describe the consequences of a stroke.

### Statistical Analysis

#### Baseline Cluster Analysis

To define stroke severity, the study population at baseline was stratified based on impairments, activity limitations, and demographics using 2-step cluster analysis. Cluster analysis is a robust technique can be used to identify homogenous subgroups that share similar features and clinical characteristics. An initial preclustering of a large data set into smaller partitions was conducted, and the preclusters were then used as inputs to be sorted into the final clusters by using hierarchical techniques on the basis of log-likelihood distance. The mixed-type data was processed by assuming independent distributions for continuous and categorical variables within the clusters.^17^ The clustering performance was evaluated based on Schwarz Bayesian information criterion.

Post hoc comparison was conducted to compare clinical characteristics of each identified cluster. Pearson χ^2^ and Fisher exact test were used for nominal variables and the Cochran Armitage test for ordinal variables, as external validation. An independent *t* test was used for comparing parametric variables and the Mann-Whitney *U* test for nonparametric variables. *P* values for multiple comparisons were adjusted using Holm-Bonferroni corrections. Missing data (2.5% of the total data) was imputated, using an iterative imputation based on a random forest algorithm trained on observed values of a data matrix to predict the missing values and proceed iteratively.^18^ The imputation method is sufficient for managing mixed types of missing data including continuous and categorical variables. Missing data imputation was conducted before the cluster analysis.

#### Longitudinal Analysis

Patients with loss to follow-up equal to ≥2 missed visits or missing >30% of values were further excluded before longitudinal analysis. A random-coefficient model was applied to analyze time effects and compare the rate of change in functional mobility over time between different levels of stroke severity by fitting a group and time interaction.^19^ Age, stroke severity, cognition, time, quadric time and interactions between time, quadric time, and severity were included as fixed effects. Individual-level and an interaction of subject, time, and quadric time were used as the random effects. Predicted individual response to functional mobility overtime was visualized using response-profile model.^20^ Age was dichotomized into 2 sublevels: age <75 and age ≥75 years. A subanalysis was conducted to investigate functional mobility changes among age groups using repeated-measures of variance and a paired *t* test. Continuous variables were standardized before the analysis. The statistically significance level was defined as *P* <0.05.

## Results

One hundred forty patients were recruited to the study. Data from 5 patients were excluded due to loss at baseline and were withdrawn from the study.^21^ One-hundred-thirty-five patients (median age 76 years, range 37–96, 52 females [39%], Table 1) were included at baseline. An additional 44 patients were excluded owing to loss to follow-up (n=20), withdrawal (n=18), or new stroke or other diseases that affect motor function (n=6). A total of 91 participants with 455 measurements were included in the longitudinal analysis. There were no statistically significant differences in age, sex, or neurological deficit severity between the patients who were excluded and the participants included in the longitudinal analysis.

**Table 1.**
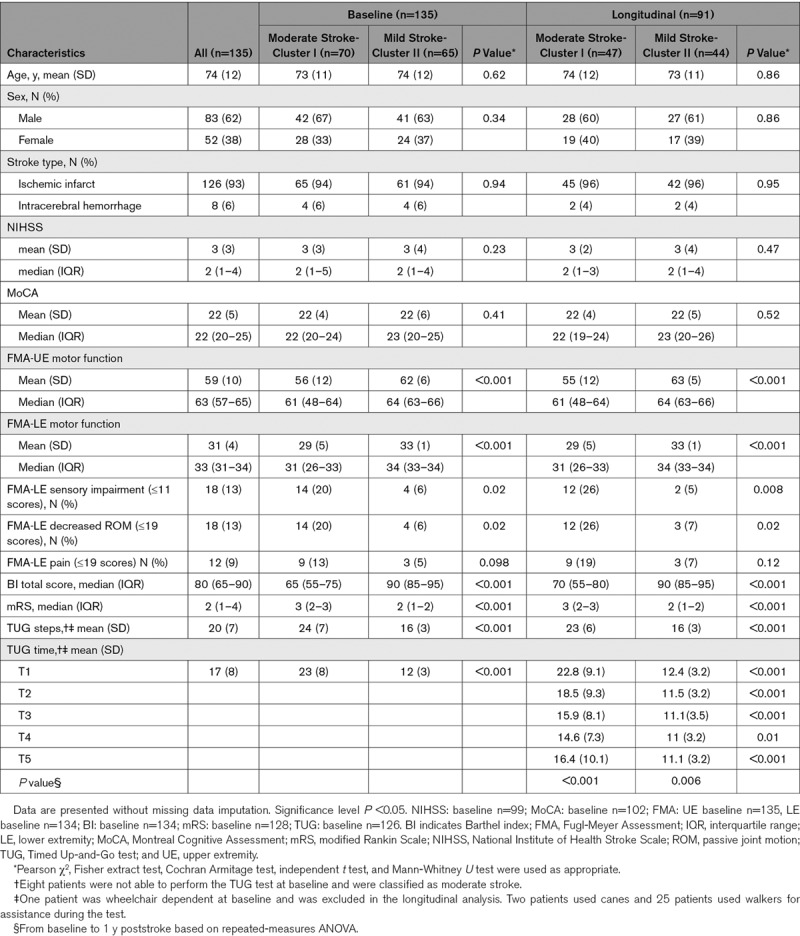
Clinical Characteristics and Demographics of Patients at Baseline and in Longitudinal Analysis

### Baseline Evaluation Based on Cluster Analysis

**Figure 1.**
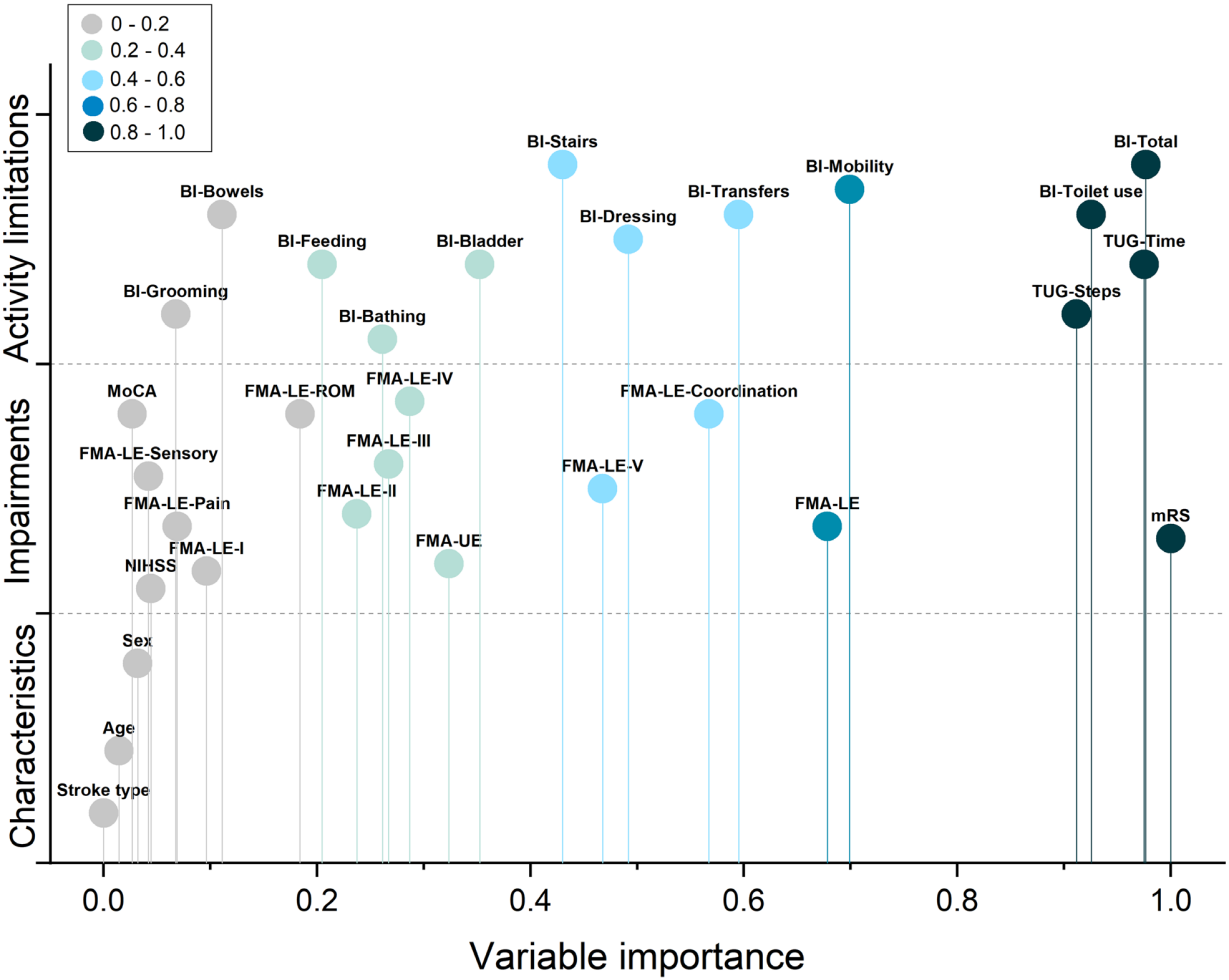
**Importance of included variables in the cluster analysis is shown.** The variables with higher values indicate more contribution for stratifying that clusters from the others. BI indicates Barthel Index; FMA, Fugl-Meyer Assessment; FMA-LE, FMA lower extremity; FMA-LE-I, reflex activity; FMA-LE-II, FMA-LE volitional movement within synergies; FMA-LE-III, FMA-LE volitional movement mixing synergies; FMA-LE-IV, FMA-LE volitional movement with little or no synergy; FMA-LE-ROM, FMA-LE passive joint motion; FMA-LE-V, FMA-LE normal reflex activity; FMA-UE, FMA upper extremity; MoCA, Montreal Cognitive Assessment; mRS, modified Rankin Scale; NIHSS, National Institute of Health Stroke Scale; and TUG, Timed Up-and-Go test.

The clinical variables included in the baseline cluster analysis are shown in Figure 1. The cluster algorithm stratified the following 2 distinct groups of stroke severity based on impairments and activity limitations: moderate stroke (70 of 135 patents, 52%) and mild stroke (65 patients, 48%). The variables that contributed most to determine clusters were TUG time, TUG steps, modified Rankin Scale, total BI scores, and capacity to use the toilet independently as assessed using the BI subitem (Table 1 and Figure 1).

The moderate stroke group was characterized by noticeably reduced function (median [interquartile range] modified Rankin Scale score, 3 [2–3]; mean [SD] TUG time, 22.8 s [8.4]; mean [SD] Fugl-Meyer Assessment, LE, 29.2 [4.9]; Table 1), and moderate activity limitations (median [interquartile range] total BI scores, 65 [55–75]).

The mild stroke group was characterized by slightly reduced function (median [interquartile range] modified Rankin Scale, 2 [1–2]; mean [SD] TUG time, 12.3 s [3.1]; mean [SD] Fugl-Meyer Assessment, LE, 33.4 [1.1]; Table 1), and mild or no activity limitations (median [interquartile range] total BI scores, 90 [85–95]). Details of the cluster distributions for each variable of impairments and activity limitations are presented in the Figures in the Data Supplement (Table 1).

### Prediction of Longitudinal Progression in Functional Mobility

Of the 91 patients included in the longitudinal analysis, 47 patients (52%) had moderate stroke, and 44 (48%) had mild stroke (Table 1). There were significant differences in TUG time between mild and moderate stroke groups at 5 days after onset, within 24 hours after discharge, 1 month after discharge, and 3 months, and 1 year after poststroke.

**Figure 2.**
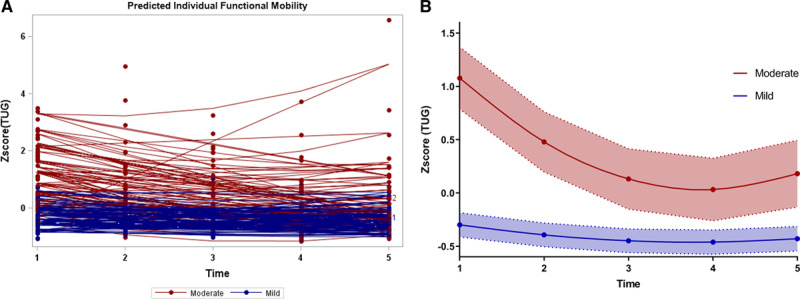
**Longitudinal progression in functional mobility after controlling age and cognition.** **A**, Predicted individual response in functional mobility over time. **B**, The cubic spline of predicted functional mobility with 95% CI over time. T1=5 d after onset; T2=within 24 h after discharge; T3=1 mo after discharge; T4=3 mo poststroke; T5=1 y poststroke. TUG indicates Timed Up-and-Go test.

Prediction of longitudinal functional mobility, for individuals and compared between the mild and moderate stroke groups over time, after controlling for age and cognition, is shown in Figure 2. Age (standardized β, 0.57, *P*<0.001, age <75 as reference, Table 2), severity (standardized β, 1.89, *P*<0.001, mild stroke as reference), and interactions between severity and time effects were associated with longitudinal changes in functional mobility. Cognition was noted as a nonsignificant factor for longitudinal progression (standardized β, –0.02, *P*=0.22; Figure 2).

**Table 2.**
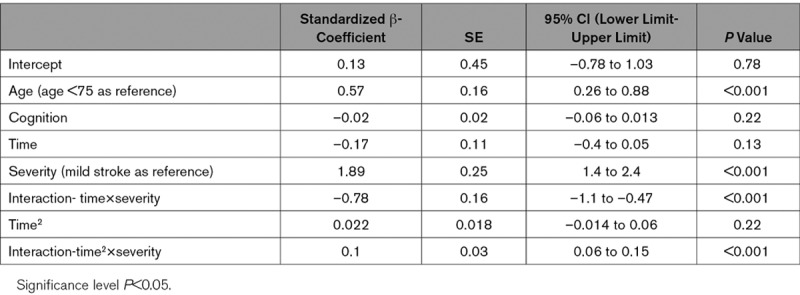
Multivariate Regression Model for Longitudinal Trend of Functional Mobility During the First Year Poststroke (n=91)

### From Baseline to 1-Year Poststroke

The moderate stroke group improved functional mobility from baseline to 1 year poststroke, after controlling for age and cognition (mean difference in TUG time, –6.4 s; least-squares [LS] mean difference, 0.87, [95% CI, 0.53–1.21], adjusted *P*<0.001). In the subanalysis, both age groups improved their functional mobility: patients with age <75 improved by 7.2 s in TUG time (*P*<0.001), and patients with age ≥75 years improved by 5.8 s (*P*=0.011).

**Figure 3.**
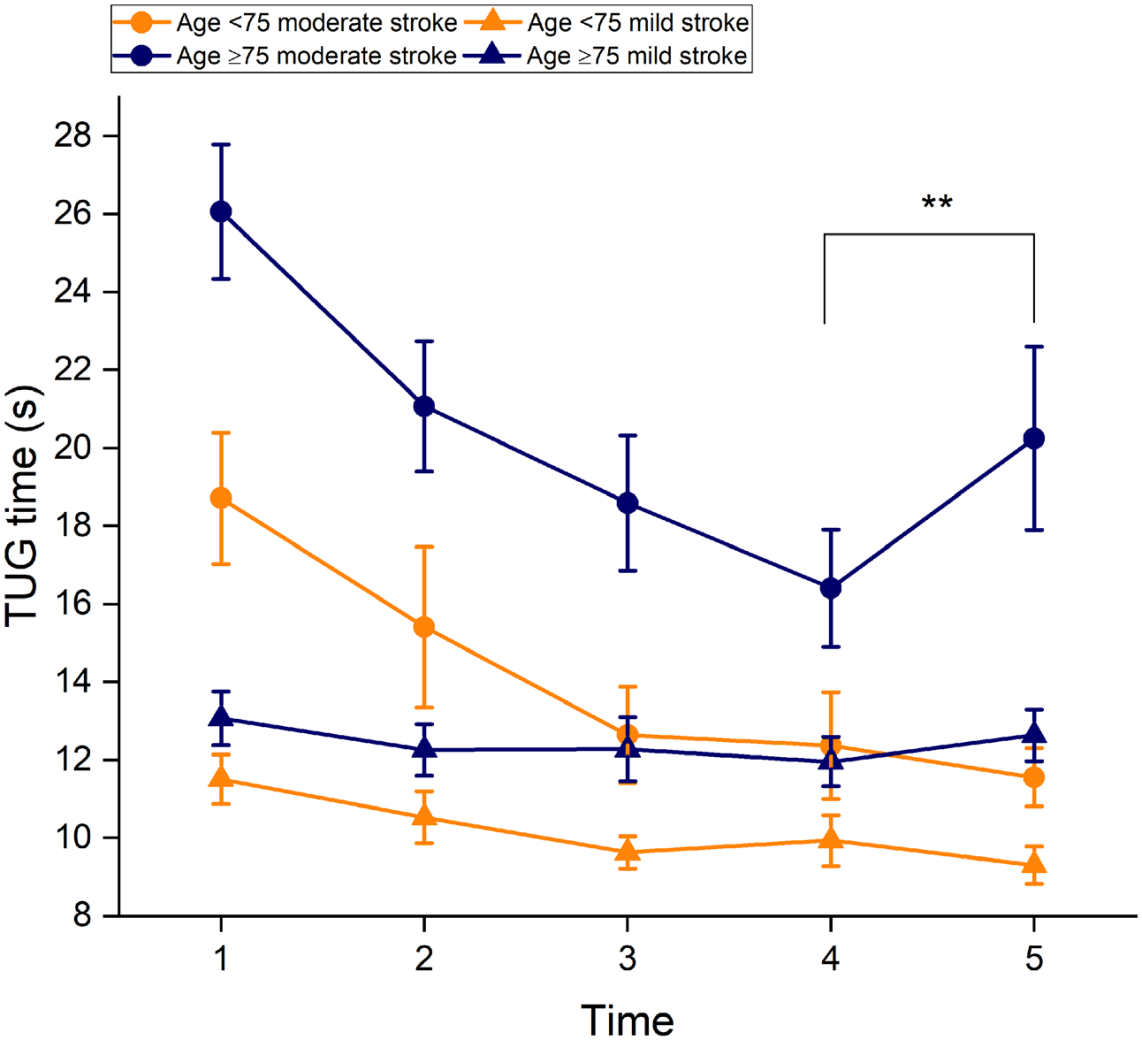
**The mean Timed Up-and-Go test (TUG) time among different age groups across 5 time points.** T1=5 d after onset; T2=within 24 h after discharge; T3=1 mo after discharge; T4=3 mo poststroke; T5=1 y poststroke. **P<0.001, comparison between T4 and T5.

In patients with mild stroke a tendency to improve was evident from baseline to 1 year poststroke, but, this was not statistically significant (LS mean difference, 0.15 [95% CI −0.22 to 0.55], adjusted *P*=0.77). In the subanalysis, patients with mild stroke age <75 at 1 year significantly improved their functional mobility (a decrease of 2.2 s in TUG time, *P*=0.003, Figure 3), compared with baseline.

Forty-one patients (87%) with moderate stroke improved in the number of steps during the TUG test at 1 year poststroke, compared with baseline, 5 worsened (11%), and one remained unchanged. Of the mild stroke group, 31 (70%) improved in the number of steps at 1 year, 6 (14%) did not change, and 7 worsened (16%).

### A Maximum Rate of Improvement During the First 3 Months

The difference in functional mobility between the 2 groups was largest at baseline and declined over time. At 3 months poststroke, the difference had diminished and then began to increase again to 1 year. The moderate stroke group had a greater improvement rate compared with the mild stroke (standardized β, 0.1, *P*<0.001, Table 2).

Patients with moderate stroke had a maximum rate of improvement in functional mobility at 3 months, compared with baseline (improved 8.2 s in TUG time, LS means difference, 1.02 [95% CI, 0.7–1.3], adjusted *P*<0.001). For mild stroke, a trend of maximal improvement rate occurred at the first month after discharge, but again no statistical significance was found (LS means difference, 0.16 [95% CI, −1.56 to 0.49], adjusted *P*=0.59).

### A Decline From 3 Months to 1 Year

In total, 55 patients (60%) displayed decreased functional mobility from 3 months to 1 year poststroke. Functional mobility worsened significantly in moderate stroke from 3 months to 1 year poststroke (12% increase in TUG time, LS mean difference, –0.23 [95% CI, –0.41 to –0.05], adjusted *P* =0.025). In the moderate stroke group, a decline was found in 31 patients (66%). Of these patients, 67% were aged ≥75. In the subanalysis, patients aged ≥75 declined significantly after 3 months (23% increase in TUG time, *P* <0.001).

No significant decline was found in mild stroke from 3 months to 1 year poststroke, regardless of age group (Figure 3).

## Discussion

In the present study, mild and moderate stroke groups were stratified using cluster analysis according to a wide range of variables of impairments and activity limitations describing 2 major domains of functioning and disability in stroke. The longitudinal progression of functional mobility differs between patients with moderate and mild stroke. Patients with moderate stroke improved their functional mobility at 1 year poststroke and had a maximum rate of improvement during the first 3 months poststroke. However, patients with moderate stroke, especially those who were aged ≥75, had a significant decline in functional mobility (12% increase in TUG time) between 3 months and 1-year poststroke. No significant improvement was found in mild stroke. Younger patients had better functional mobility.

The TUG time in our patients was, in general, longer than for healthy adults older than 70 years (reference interval of 9.4–11.3 s)^22^ across the 5 time points. A change of 2.9 s in TUG time is considered to be the minimal detected change.^23^ In this study, an improvement of 6.4 s in TUG time (a decrease of 30%) in moderate stroke noted at 1 year was therefore considered to be a clinically relevant change. Despite this, the mean TUG time at 1 year poststroke was above 16 s. This indicates a high risk of falling in patients with moderate stroke as a cutoff of 15 s in TUG time was previously suggested for a risk of falling.^24^ This finding highlighted the fact that patients with moderate stroke, especially those aged ≥75 years, may require an additional assessment of fall risk at 3 months. Although no significant improvement was found in mild stroke, an increased risk of falling has been demonstrated to be associated with a shorter TUG time after discharge.^25^ This was explained by the fact that increased mobility in mild stroke with early ambulation can increase the risks of falling.^25^

Progress in functional mobility was statistically significantly different in moderate and mild stroke. At baseline, relatively good functional mobility was observed in mild stroke, and this continued with a slight improvement of functional mobility 1 month after discharge. For moderate stroke, a significant improvement was found within 3 months. Most gain in motor recovery was shown during the first 3 months poststroke.^26^ As individual functional mobility is highly dependent on lower-limb function to complete independent walking, turning and transfer, this pattern is not surprising. Other similar recovery patterns were also noted in functional outcomes during the first 3 months, such as gait and static balance.^27,28^ Apart from TUG time, a majority of patients with moderate stroke improved the number of steps at 1 year of poststroke, compared with baseline, and this might to some extent reflect an improvement in turning ability.

The decline after 3 months observed in moderate stroke, especially with age ≥75 years, is in line with previous studies.^3,5^ One study reported a similar decrease of functional mobility from 3 months to 1 year in patients aged ≥80.^3^ Deterioration in mobility was also reported in 21% of the patients from 1 to 3 years poststroke,^5^ and approximately 43% of patients at 1 year.^4^ The present findings confirm what previous studies have found and demonstrated that a decline in functional mobility after 3 months poststroke is common. This coincides with the time when rehabilitation usually ends and the effect of spontaneous neurological recovery diminishes. Therefore, it seems that long-term rehabilitation is needed to achieve continuous improvement in functional mobility and to reduce mortality.^29^ Patients with higher age, moderate impairments and activity limitations such as inability to use the toilet independently have particular needs. The rehabilitation regimen, in terms of volume and dose, should be desirable to be better specified and tailored to older patients with moderate stroke.

Unexpectedly, cognition was not significantly associated with functional mobility changes over time in this study. Cognition was one of the most significant factors found to be responsible for functional mobility decline in many studies.^5,6^ One possible explanation is the fact that TUG performance might require a certain level of cognition but the performance might not be dominated by cognition. Another explanation could be that patients with some extent of cognitive decline may be unaware of their surroundings and self-impairment, and this might lead them to walk faster. Some previous studies also found that TUG time did not significantly differ between patients with mild cognitive impairment and without cognitive impairment.^30,31^

The strengths of this study are that the sample size was relatively large and patients were included in the acute stroke phase across 5 time points. This study is unique as it takes multidimensional parameters, such as impairments and activity limitations, into account to develop a time-dependent model for predicting the recovery of functional mobility, rather than using one single cutoff value of function assessment. It reflects a more accurate description of patients’ functioning and disability following the framework of the International Classification of Functioning.^32^ The use of a random-coefficient model in the study allowed the relationship between time and recovery of functional mobility to be explored while considering within-subject and between-subject variability. The model was developed by incorporating a nonlinear time effect that provides a more valid interpretation of recovery prediction as well as individual changes in functional mobility.

The knowledge provided by the study could help to deliver correct information for patients regarding their expected recovery after stroke, and enable early discharge planning in certain patient groups. Mobility aids as well as further rehabilitation may also need to be considered in patients with moderate stroke after the first 3 months poststroke. And a fall risk assessment at 3 months could lead to an early fall prevention. The study findings might contribute to future randomized controlled trials with the aim to investigate intervention effects for continuous gain after 3 months onset on different stroke severities.

One limitation is that the study contained few patients with stroke with severe deficits. The study population had, in general, better function and activity level than other study populations. The generalizability of the study results may, therefore, be limited to patients with mild or moderate stroke. Although cluster analysis was able to identify 2 distinct subgroups based on various deficit levels, additional variables (ie, neuropsychological factors or lower-limb muscle strength), and more patients with severe deficits may be needed to further refine the clusters. The study findings should be validated in another data set.

## Conclusions

The recovery of functional mobility differs between mild and moderate stroke. Patients with moderate stroke improved their functional mobility at 1 year poststroke but experienced a decline in functional mobility between 3 months poststroke and 1 year, especially patients over the age of 75 years. Functional mobility in the moderate stroke group had maximum improvement at 3 months poststroke. These findings suggest that long-term rehabilitation may be desirable to maintain gains in functional mobility and that elders have greater needs.

## Acknowledgments

We gratefully acknowledge all patients who participated in the study and health professionals who helped with the data collection.

## Sources of Funding

This study was funded in part by grants from the Swedish Science Council (VR2012-3523 and VR2017-00946), the Health & Medical Care Committee of the Regional Executive Board of the Region Västra Götaland, King Gustaf V’s and Queen Victoria’s Freemasons´ Foundation, the Swedish National Stroke Association, Agneta Prytz-Folke’s Gösta Folke’s Foundation, FRF foundation, and the Promobilia foundation. The study was financed by grants from the Swedish state under an agreement between the Swedish government and the county councils, the ALF agreement (ALFGBG-718711).

## Disclosures

None.

## Supplementary Material


