# Supplementary material for: Predicting Longitudinal Progression in Functional Mobility After Stroke: A Prospective Cohort Study
Source: Stroke. 2020 Jun 17;51(7):2179–87. doi: 10.1161/STROKEAHA.120.029913 (PMC7306259; doi:10.1161/STROKEAHA.120.029913)
Supplement: Supplementary file 1 [file str-51-2179-s001.pdf]

SUPPLEMENTAL MATERIAL

Predicting Longitudinal Changes in Functional Mobility After Stroke: A Prospective Cohort Study

Cover title: Changes in functional mobility after stroke

Dongni Buvarp MD Ph.D,[1] Lena Rafsten, PT MSc,[1,2] Katharina S Sunnerhagen, MD Ph.D[1,2]

[1]Rehabilitation Research Group, Department of Clinical Neuroscience, Institute of Neuroscience and Physiology, Sahlgrenska Academy, University of Gothenburg and

[2]Sahlgrenska University Hospital, Gothenburg, Sweden

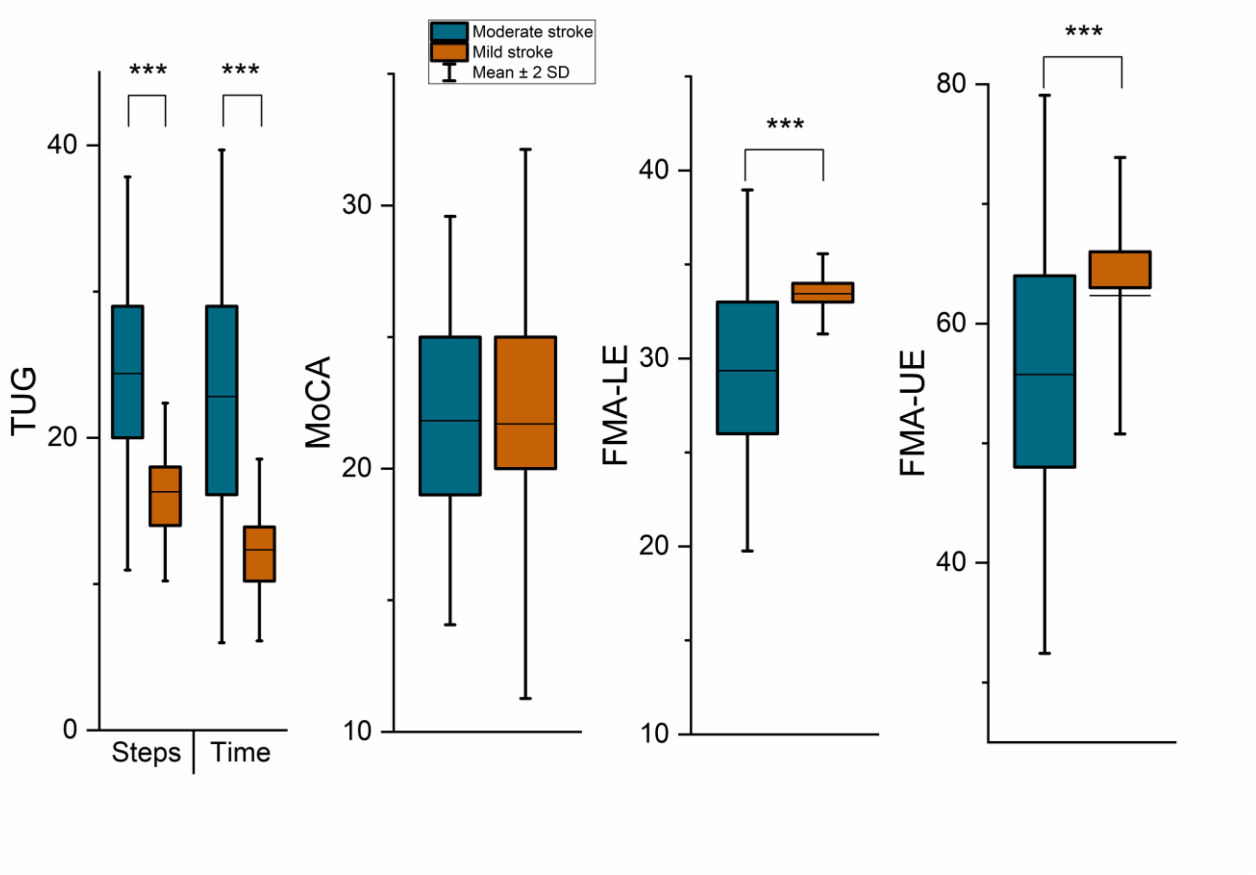

Supplementary Figure I. Distribution of impairments between clusters is shown. SD, standard deviation; TUG, Timed Up-and-Go test; MoCA, Montreal Cognitive Assessment; FMA, Fugl-Meyer Assessment; UE, upper extremity; LE, lower extremity.*** $P < 0.001$

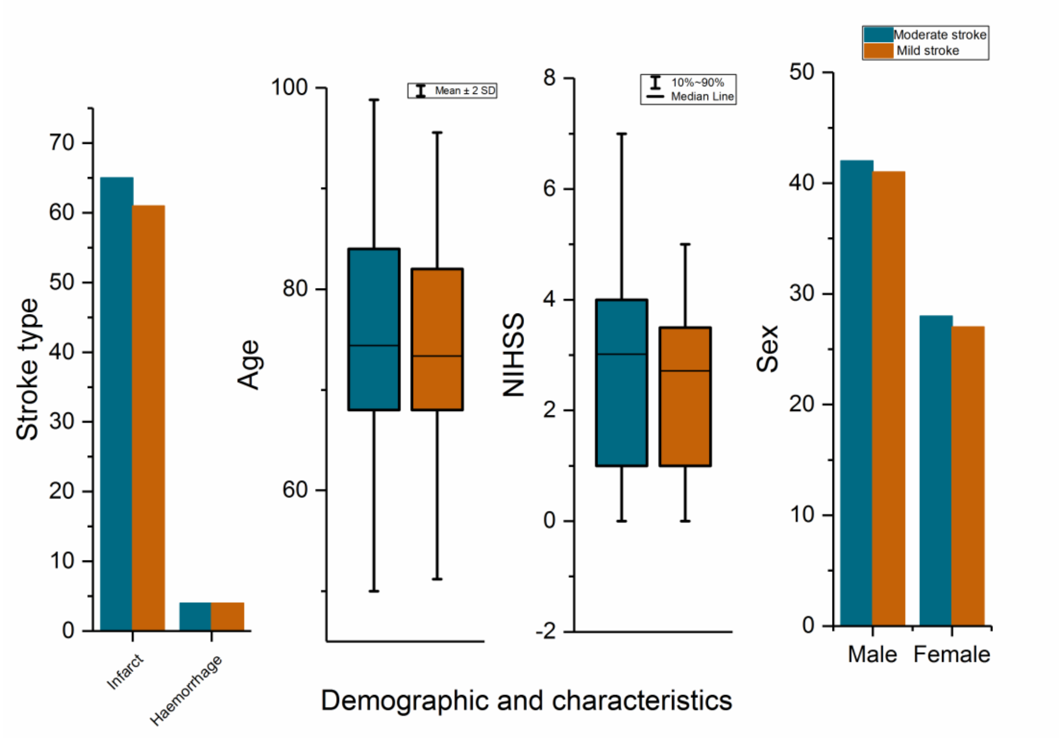

Supplementary Figure II. Distribution of demographic and characteristics between clusters is shown. NIHSS, National Institute of Health Stroke Scale;

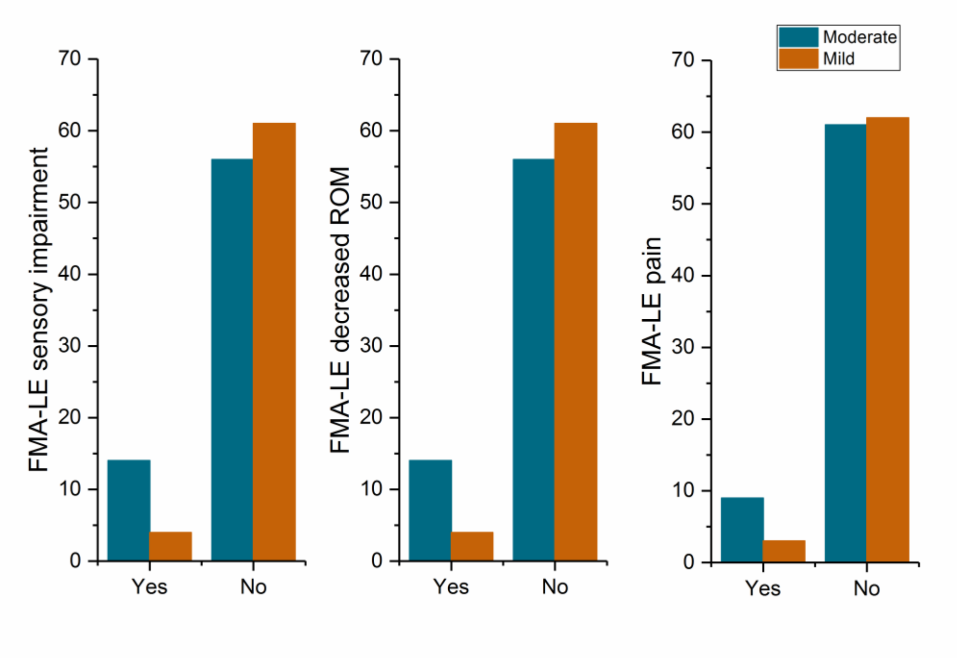

Supplementary Figure III. Distribution of impairments in lower-limb between clusters is shown. FMA, Fugl-Meyer Assessment; LE, lower extremity.

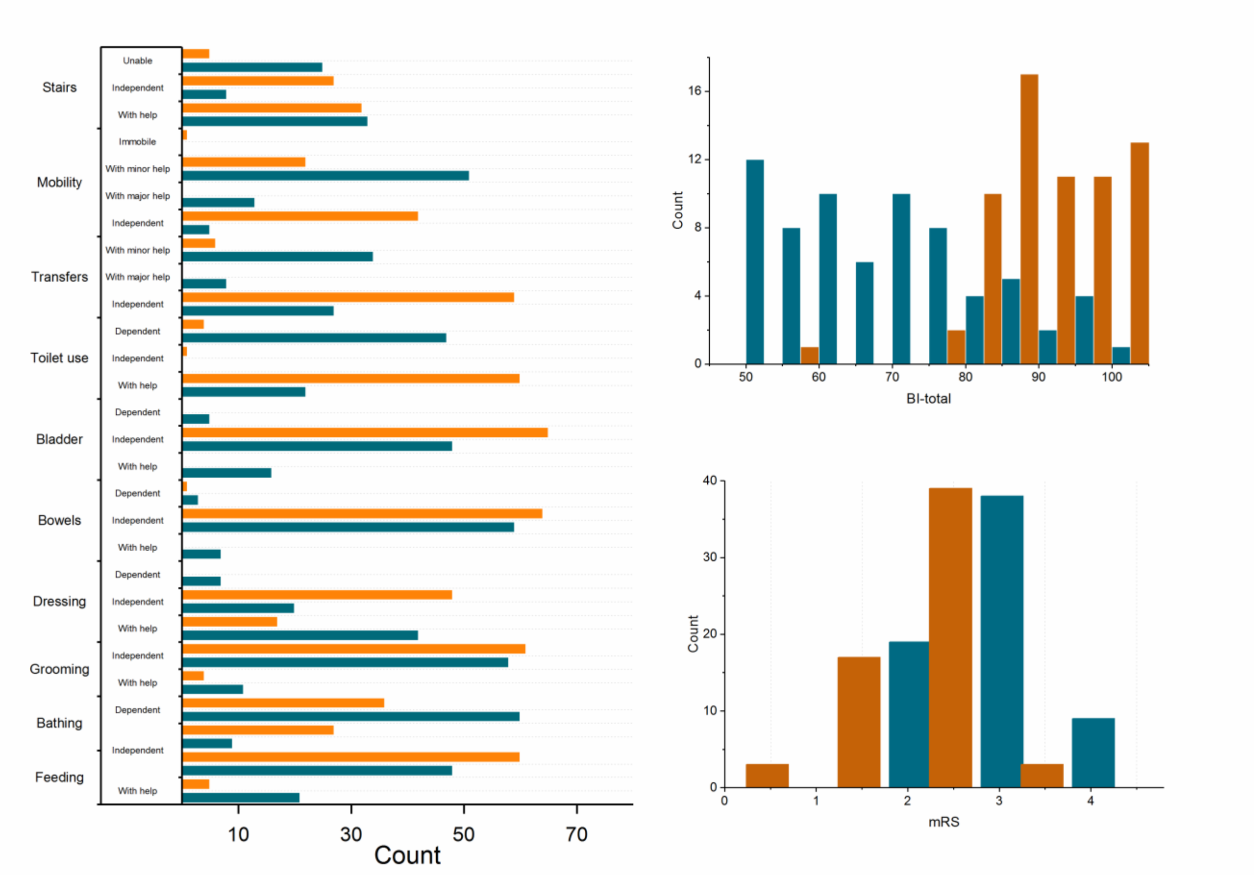

Supplementary Figure IV. Distribution of activity limitations between clusters is shown. BI, Barthel index; mRS, modified Rankin Scale;

Statistics analysis

Baseline cluster analysis for processing mixed type data was conducted in R 3.6.1 (R Foundation for Statistical Computing, Vienna, Austria). Missing values was imputed using package ‘MissForest’ in R version 3.6.1 (R Foundation for Statistical Computing, Vienna, Austria). A random-coefficient model was developed using PROC MIXED in SAS version 9.4 (SAS Institute, Inc, Cary, NC).
